# The Effect of an Adsorbent Matrix on Recovery of Microorganisms from Hydrocarbon-Contaminated Groundwater

**DOI:** 10.3390/microorganisms9010090

**Published:** 2021-01-01

**Authors:** Nicole M. Taylor, Courtney R. A. Toth, Victoria Collins, Paolo Mussone, Lisa M. Gieg

**Affiliations:** 1 Petroleum Microbiology Research Group, Department of Biological Sciences, University of Calgary, 2500 University Drive NW, Calgary, AB T2N 1N4, Canada; nicole.taylor1@ucalgary.ca; 2 Department of Chemical Engineering and Applied Chemistry, University of Toronto, 200 College Street, Toronto, ON M5S 3E5, Canada; courtney.toth@utoronto.ca; 3 Applied BioNanotechnology Industrial Research Chair Program, Northern Alberta Institute of Technology, 11762-106 Street, Edmonton, AB T5G 2R1, Canada; ccollins@nait.ca (V.C.); pmussone@nait.ca (P.M.)

**Keywords:** hydrocarbon biodegradation, bioremediation, groundwater, microcosm, Tenax-TA

## Abstract

The microbial degradation of recalcitrant hydrocarbons is an important process that can contribute to the remediation of oil and gas-contaminated environments. Due to the complex structure of subsurface terrestrial environments, it is important to identify the microbial communities that may be contributing to biodegradation processes, along with their abilities to metabolize different hydrocarbons in situ. In this study, a variety of adsorbent materials were assessed for their ability to trap both hydrocarbons and microorganisms in contaminated groundwater. Of the materials tested, a porous polymer resin (Tenax-TA) recovered the highest diversity of microbial taxa in preliminary experiments and was selected for additional (microcosm-based) testing. Oxic and anoxic experiments were prepared with groundwater collected from a contaminated aquifer to assess the ability of Tenax-TA to adsorb two environmental hydrocarbon contaminants of interest (toluene and benzene) while simultaneously providing a surface for microbial growth and hydrocarbon biodegradation. Microorganisms in oxic microcosms completely degraded both targets within 14 days of incubation, while anoxically-incubated microorganisms metabolized toluene but not benzene in less than 80 days. Community analysis of Tenax-TA-associated microorganisms revealed taxa highly enriched in sessile hydrocarbon-degrading treatments, including *Saprospiraceae*, *Azoarcus*, and *Desulfoprunum*, which may facilitate hydrocarbon degradation. This study showed that Tenax-TA can be used as a matrix to effectively trap both microorganisms and hydrocarbons in contaminated environmental systems for assessing and studying hydrocarbon-degrading microorganisms of interest.

## 1. Introduction

Monoaromatic hydrocarbons found in polluted environments, such as benzene and toluene, are chemically unreactive due to their non-polar nature and aromatic character [1]. Many microorganisms in hydrocarbon-contaminated environments can metabolize aromatic hydrocarbons as their carbon and energy sources, thus contributing to site remediation processes. The microbial degradation of aromatic hydrocarbons is well characterized under aerobic conditions [1,2,3]. Anaerobic hydrocarbon biodegradation pathways have also been characterized to varying extents in recent years and involve different activation mechanisms in the absence of oxygen [4]. While many polycyclic aromatic hydrocarbons are recalcitrant under anoxic conditions (with a few notable exceptions [5,6]), several studies have shown that monoaromatic hydrocarbons such as toluene can be metabolized when coupled with available anaerobic electron acceptors or under methanogenic conditions [7,8,9,10,11]. Benzene biodegradation has also been reported under a variety of anoxic conditions [12,13,14,15,16,17,18], though the mechanism(s) catalyzing its degradation are still not fully understood.

The heterogeneity and diversity of subsurface groundwater environments constitute a major challenge in assessing the in situ role of microorganisms for hydrocarbon remediation. Most degradation studies focus heavily on the activity of planktonic cells. However, sessile or biofilm-associated cells are likely more important in subsurface environments. Microorganisms in groundwater ecosystems can be attached to solid matrices or exist planktonically; several reports have estimated that only a small fraction (0.06–0.22%) remains unattached [19], aligning with the recent estimate that up to 80% of all microbial life across major global habitats is surface attached [20]. In nature, communities of diverse microorganisms typically work together in biofilms or aggregates to carry out metabolic processes (including hydrocarbon degradation) and nutrient cycling either synergistically or syntrophically [21,22], thus capturing these aggregate communities from different environments can allow for a deeper understanding of their ecosystem functioning.

Collecting groundwater samples from monitoring wells is the simplest and most common way to identify microorganisms and their potential activities in fuel-contaminated sites [23,24,25]. This method of sample collection primarily targets planktonic cells, and therefore may greatly underestimate the microbial communities inhabiting groundwater systems given the presumed predominance of surface-attached organisms. Obtaining core samples from the subsurface is a more ideal method to capture surface-attached cells. However, this can be logistically challenging and costly. One alternative to these methods is to use samplers containing adsorptive solid support material that can be emplaced into groundwater monitoring wells to collect microorganisms that are prone to form biofilms. This approach has been reported in several studies which have shown the efficacy of using solid support materials to sample microorganisms in subsurface environments that may be contributing to hydrocarbon metabolism [26]. Materials such as granular activated carbon [25] and Bio-Sep^®^ beads (comprised of activated charcoal in a polymer matrix, [27]) have been successfully used to sample microorganisms from hydrocarbon-contaminated groundwater environments for microbial community member identification [24,28,29,30]. Further, materials such as the Bio-Sep^®^ beads have been incorporated into commercially available Bio-Trap^®^ samplers (Microbial Insights, Knoxville, TN, USA). Increased appreciation of the role of surface-attached microorganisms highlights the need to assess the impact of these solid supports in groundwater ecosystems.

**Figure 1:**
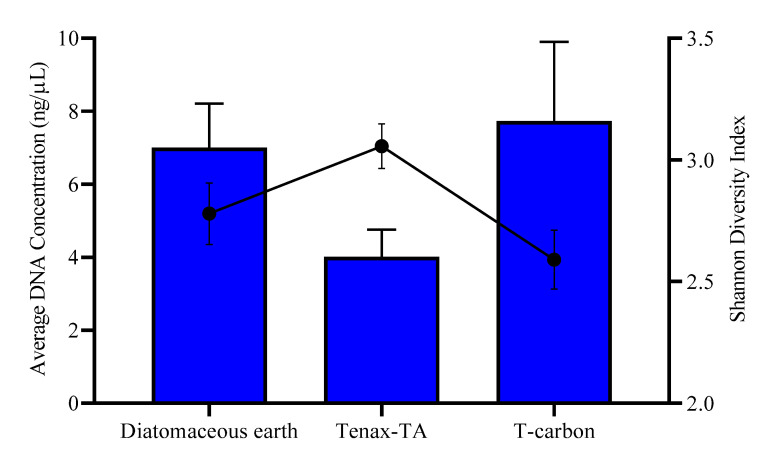
Average DNA recoveries from various trap matrices (blue bars) initially tested in groundwater samplers, and the associated average Shannon diversity indices (black points) of the microbial communities analyzed through 16S rRNA gene sequencing and R (vegan). Error bars represent the standard error of the mean of 18 replicates per treatment.

**Figure 2:**
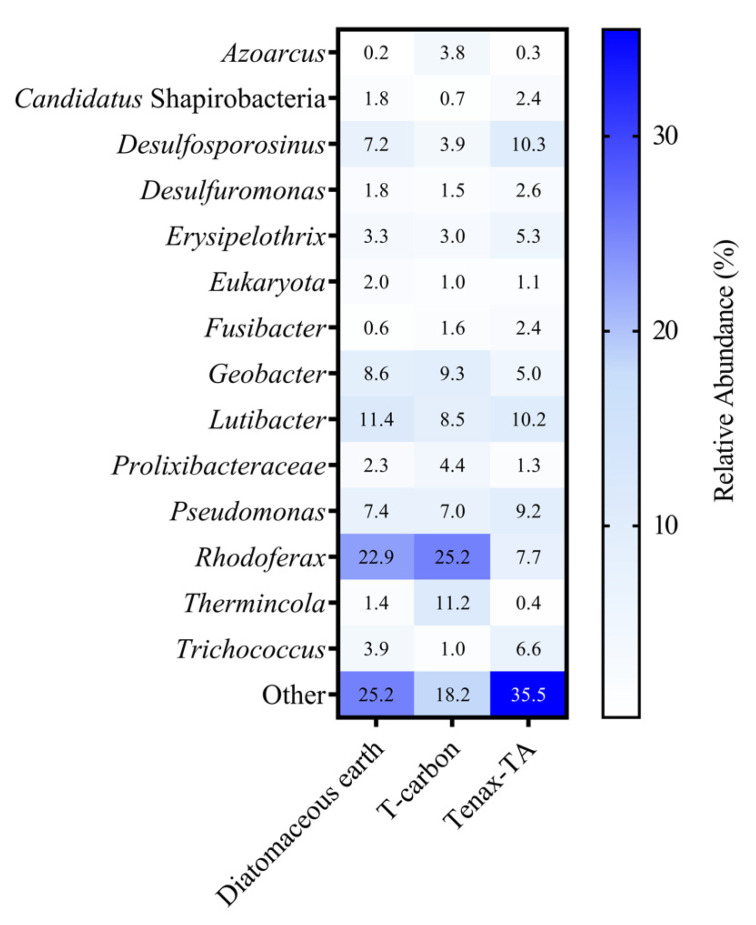
Microbial community composition of field samples collected in 2017. The percent relative abundances of the top ten taxa recovered from each material from all sampling dates (August, September, and October) and depths (3, 4, and 5 m) surveyed were averaged and are displayed. All other taxa (ASVs less than 0.2% relative abundance) are grouped as ‘Other’.

In this study, we sought to test a variety of other high-surface-area materials for their ability to trap and enrich putative fuel-degrading microorganisms from hydrocarbon-contaminated groundwater. This work is part of a larger study that is developing an “all-in-one” prototype groundwater sampling device equipped with multiple components for measuring different parameters of contaminated groundwater. As such, the sampler includes a component to collect and identify surface-attached microorganisms. Following a preliminary sorption test with soil (Table S1) and a subsequent field-based experiment (Figure 1 and Figure 2, Table S2), one commercially available material, Tenax^®^ trapping agent (herein referred to as Tenax-TA), was found to capture the most microbial diversity. This sorptive polymer resin was then further evaluated through a series of laboratory microcosm experiments to assess whether the trapping agent could capture microorganisms capable of degrading common groundwater contaminants (benzene and toluene) under various electron-accepting conditions. We also sought to compare how microorganisms grown in this sessile manner differed from the corresponding planktonic communities. Our findings can be applied to future studies to better characterize passively trapped hydrocarbon-degrading microorganisms in groundwater environments.

## 2. Materials and Methods

### 2.1. Survey of Adsorbent Materials

In preliminary microcosm tests, five adsorbent materials (zeolite, activated carbon, Mat540, diatomaceous earth (DE), and T-carbon (TC)) were surveyed for their use as microbial traps in soil and groundwater environments. Specifications for each adsorbent are included as Supplementary Materials (see Table S1). Nylon pouches (60 μm mesh, approximately 1.5 × 2.0 cm) containing the porous trapping agents (125 ± 5 mg) were added to microcosms containing 5 g soil and 30 mL sterile DI water to simulate an environmental sampling well. This initial soil sample was collected from just below the water table at a hydrocarbon-contaminated aquifer location near Saskatoon, SK, Canada. After six days of incubation at room temperature, the trapping agents were extracted for DNA, described below, and recoveries were quantified using Qubit fluorometry (Thermo Fisher Scientific, Waltham, MA, USA). Based on greater DNA recoveries (Table S1), DE and TC were selected for field testing in a different hydrocarbon-contaminated aquifer also located near Saskatoon, SK, Canada. A third material, Tenax-TA (60–80 mesh, 25550; Restek, Bellefonte, PA, USA), was also included in this field trial given its known ability to adsorb hydrocarbons and its moderate sorption surface area (35 to 40 m^2^/g), which is a desirable feature to facilitate colonization by microorganisms [31,32]. The hydrocarbon sorption efficiency of Tenax-TA was verified in sterile experimental trials prepared with distilled water and known amounts of two hydrocarbons of interest: benzene and toluene (see Figure S1).

To deploy the trapping agents at the Saskatoon field site, the sorbents DE, TC, and Tenax-TA were prepared as described above). Eighteen pouches were prepared for each sorbent, for a total of 54 samplers. These adsorbent traps were then attached with nylon thread onto the prototype sampling device so that they could be suspended into a test groundwater monitoring well. Duplicate samplers were deployed directly into the groundwater aquifer at depths of 3, 4, and 5 m below ground surface. Once a month, for a total of three months, two replicates of each sorbent sampler were recovered from each experimental depth for molecular analysis. Samplers were stored immediately at −20 ℃ and transported on ice to the University of Calgary for DNA extraction and microbial community analysis, according to the methods detailed below. DNA recoveries for each pouch are reported in Table S2. As described in the Results section, the samplers containing Tenax-TA captured greater species richness, more high-quality reads, and greater microbial diversity than DE or TC. As such, Tenax-TA was chosen for further study in controlled laboratory incubations.

### 2.2. Experimental Microcosms

In a third series of experiments, we sought to further assess the performance of Tenax-TA samplers in capturing both hydrocarbons and microorganisms. The purpose of this test was to differentiate surface-attached versus planktonic microorganisms, if any, and to determine whether the use of such sorptive traps could help to stimulate hydrocarbon biodegradation under different electron-accepting conditions. Groundwater slurries (containing some sand particles) from a hydrocarbon-contaminated aquifer located near Stony Plain, AB, Canada were collected in June 2018 and served as the inoculum for new microcosm experiments. Chemical analyses revealed the groundwater to be neutral in pH (7.72 ± 0.03) and contain 1.84 ± 0.07 mM total iron, a sulfate concentration of 0.74 ± 0.04 mM, and freshwater salinity (0.03%). No nitrate, nitrite, organic acids (such as acetate) or aromatic hydrocarbons were detected in these samples. However, previous groundwater analyses (in 2016) indicated that benzene and toluene levels in this site reached 8.39 mg/L and 0.377 mg/L [33], both exceeding Canadian maximum allowable concentrations (0.005 and 0.024 mg/L, respectively [34]). The lack of hydrocarbons detected in the present groundwater samples was likely due to volatilization that occurred during transportation and storage.

Experimental microcosms were established in triplicate using 30 mL of groundwater-sand slurry transferred anoxically under N_2_ gas into sterile 60 mL glass serum bottles, closed with butyl rubber stoppers (Bellco Glass Inc., Vineland, NJ, USA) and sealed with aluminum crimps, creating a total of 120 microcosms (Table 1). Half of the microcosms received a nylon pouch containing Tenax-TA beads (prepared as described above) which were suspended from the rubber stopper using nylon thread (as shown in Figure S2). These sealed microcosms containing Tenax-TA were sterilized by autoclaving (treatment at 121 °C does not affect the properties of Tenax-TA, [31,32]) before addition of the groundwater slurry. The remaining half were not provided a pouch to determine any selective pressures that might be exerted by Tenax-TA. Microcosms were amended with 0.5 μL each of 99.8% anhydrous benzene and toluene (Sigma Aldrich, Oakville, ON, Canada), representing 5.6 μmoles and 4.7 μmoles, respectively. Select bottles also received exogenous electron acceptors as per Table 1. Further established were a set of heat-killed controls (prepared by autoclaving groundwater three times with 24 h intervals to ensure the sterilization of spore-forming organisms) and hydrocarbon-free (unamended) controls to ensure that all variations of possible experimental outcomes were accounted for. All microcosms were incubated at room temperature (21–22 °C) in the dark for 80 days.

### 2.3. Analytical Procedures

Benzene and toluene were measured weekly by headspace sampling and gas chromatography–flame ionization detection (GC-FID) over 80 days of incubation and quantified based on calibration curves prepared from known concentrations of these hydrocarbons [35,36]. Operating parameters for hydrocarbon [37] and methane detection [10] by GC-FID, as well as carbon dioxide and oxygen detection by gas chromatography equipped with thermal conductivity detection (GC-TCD) [38], have been described in detail elsewhere. Nitrate and sulfate concentrations were measured using high performance liquid chromatography (HPLC) ion exchange with an acetonitrile/borate/gluconate mobile phase, UV/Vis and conductivity detection [39]. Iron(II) (the product of microbial Fe(III) reduction) was measured spectrophotometrically using the ferrozine assay [40]. Where appropriate, unpaired two-tailed Student’s *t*-tests were calculated to determine statistically significant differences between means.

### 2.4. Microbial Community Analysis by 16S rRNA Gene Sequencing

After 80 days of incubation, live microcosms were sacrificed for DNA extraction. DNA was extracted from the both the planktonic and sessile (Tenax-TA) fractions separately. Planktonic microorganisms were collected by centrifuging 5 mL of the liquid fraction at 14,000 rpm for 10 min, discarding the supernatant, and immediately proceeding with the extraction. Tenax-TA pouches were aseptically transferred to sterile tubes, cut in half and vortexed for 1 min with sodium phosphate buffer to detach the microbial cells from the Tenax-TA matrix. For DNA extraction, a modified FastDNA™ SPIN Kit for Soil (MP Biomedicals, Santa Ana, CA, USA) protocol was used. This involved treatment with proteinase K (20 mg/mL; Thermo Fisher Scientific) and sodium dodecyl sulfate solution (20%) for 30 min at 50 °C after bead beating to enhance protein digestion, followed by the manufacturer’s standard protocol. Field samplers and preliminary soil microcosm test pouches were processed for DNA extraction in the same way; however, the soil microcosm tests omitted the proteinase K step (later added for improved protein digestion and DNA recovery). Extracted DNA was then subjected to a two-step PCR protocol to target the V6-V8 hypervariable region of the 16S rRNA gene as previously described [41]. Prepared samples were sequenced using the Illumina MiSeq^®^ platform (300PE) at the Alberta Children’s Hospital Research Institute (Calgary, AB, Canada). Sequenced amplicon data were processed in QIIME 2.0 (version 2019.10) using default quality control settings [42]. Low-quality reads and sequences of incorrect lengths (truncated forward reads < 280 bp, reverse reads < 260 bp) were removed and acceptable length paired reads were joined with DADA2 denoising algorithms [43] in order to sort amplicon sequence variants (ASVs). Annotation was conducted using Naïve-Bayes classifiers and the SILVA 132 16S SSU database [44]. R was used for computing statistical analysis of microbial communities (vegan, ape, and picante) and associated graphics (ggplot2). Due to highly diverse samples each containing hundreds of unique taxa, only the top five to ten most abundant taxa are reported in figures in Section 3.1 and 3.4; however, all taxa were included in diversity analyses.

### 2.5. Scanning Electron Microscopy

Some aerobically incubated Tenax-TA pouches were preserved for biofilm visualization by scanning electron microscopy, according to the fixation and dehydration method outlined by Lewandowski and Beyenal [45]. Preserved samples were loaded on carbon tape, sputtered with gold, and visualized with a TESCAN VEGA3 scanning electron microscope at the NAIT Nanotechnology lab (Edmonton, AB, Canada).

## 3. Results

### 3.1. Field Testing of Microbial Trapping Matrices

In July 2017, sampling pouches containing DE, TC, or Tenax-TA materials were deployed at three different depths into a contaminated aquifer near Saskatoon, SK, Canada. Pouches were recovered from each depth in one-month intervals for a total of three months, and two pouches per time point were extracted for DNA and their microbial community profiles analyzed by 16S rRNA gene amplicon sequencing.

Similar microbial communities were captured across all sampling dates, depths, and materials, albeit in varying relative abundances. Tenax-TA had lower average DNA recoveries than DE or TC (Figure 1). However, this material trapped more diverse taxa (measured by the Shannon index; Figure 1 and Table S2) and had a greater proportion of unique or low abundance taxa (less than 0.3%) than DE or TC (Figure 2). *Rhodoferax* dominated the microbial communities in DE and TC sampling pouches (mean relative abundances of 22.9% and 25.2%, respectively; Figure 2), while it was significantly less abundant in Tenax-TA (7.7%, *p*-values ≤ 0.01 and ≤ 0.001, respectively). Putative hydrocarbon degraders were captured by all materials, including taxa such as *Azoarcus, Desulfosporosinus, Geobacter, Pseudomonas,* and *Thermincola*. Due to superior taxonomic diversity, capture of a variety of hydrocarbon-degraders, and comparatively lower bias for *Rhodoferax* (Figure 2), Tenax-TA was chosen for further analysis in the microcosm experiments.

### 3.2. Hydrocarbon Biodegradation and Mineralization of Electron Acceptors

Here, Tenax-TA was screened for its ability to sorb hydrocarbons (and thus associate with potential hydrocarbon-utilizing microorganisms), differentiate surface-attached versus planktonic microorganisms, and whether its use in sampler traps would stimulate hydrocarbon biodegradation under different electron-accepting conditions. We began by assessing the ability of Tenax-TA to sorb aromatic hydrocarbons, compared to treatments without the traps in an abiotic experiment. Toluene (77%) and benzene (53%) were sorbed significantly within one day of hydrocarbon addition (Figure S1) compared to those without Tenax-TA (representing 100% of available hydrocarbons). This result was consistent with other studies of benzene and toluene adsorption [46].

**Figure 3:**
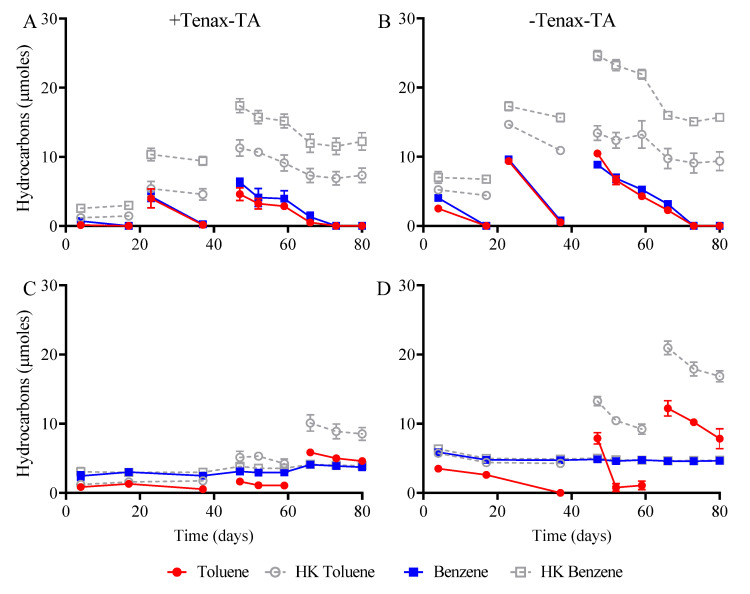
Hydrocarbon biodegradation profiles from aerobic microcosms with (**A**) and without Tenax-TA (**B**) as well as no electron acceptor (EA)-added control microcosms with (**C**) and without Tenax-TA (**D**). Toluene and benzene were both degraded in aerobic microcosms within 14 days of incubation; control microcosms experienced an initial lag of 40 days before toluene degradation was observed, after which it was completely degraded within 14–20 days. Benzene was not degraded during the monitoring period. Gaps in the plots represent depletion and re-amendment of hydrocarbons. Live and heat-killed controls (HK) were established. Error bars depict the standard error of the mean of 3 replicates.

Microorganisms in oxically prepared microcosms (O_2_) completely oxidized both toluene and benzene after approximately 14 days of incubation, which was reproducible after refeeding bottles additional hydrocarbon substrate (Table 2; Figure 3). They also demonstrated the fastest overall rate of hydrocarbon degradation (Table 3). Carbon dioxide production in aerobic microcosms with Tenax-TA was 80% higher than those without Tenax-TA (97.9 ± 0.7 compared to 55.0 ± 2.0 μmoles, respectively), a statistically significant difference (*p*-value ≤ 0.01) representing 49% and 28% of the predicted stoichiometric yield (Table 2; Table 3). The difference in this yield may be explained by conversion to biomass. In contrast, all microcosms incubated anoxically were capable of toluene degradation, but benzene metabolism was never observed during the 80 day monitoring period (Figure S3). Nitrate-reducing microcosms had the fastest rate of toluene degradation (0.2 μmoles/day; Table 3), and displayed only a slightly significant difference in the extent of nitrate reduction between Tenax-TA and Tenax-TA-free treatments (91.8 ± 5.5 and 113.2 ± 4.6 μmoles; Table 3). No nitrite was detected, suggesting that complete denitrification occurred in both Tenax-TA and Tenax-TA-free treatments. Iron(III)-reducing microcosms natively contained 32.5 ± 1.3 μmoles of Fe(II) at the outset of the experiment (Day 0). Of the predicted stoichiometric yield (Table 2; Table 3), only 7% of this amount was realized in incubations with Tenax-TA by the end of the experiment. Tenax-TA-free treatments yielded 53% of the predicted stoichiometric yield, indicating that Tenax-TA could in some way be inhibitory to iron(III) reduction or the growth of iron-reducing microorganisms in this experiment. The exact cause of these results is unknown. Interestingly, toluene degradation was unaffected (Figure S3E) by the lack of iron reduction, suggesting an alternate anaerobic respiration pathway was coupled to the mineralization of this substrate. Sulfate-reducing treatments did not display a significant difference in sulfate reduction between Tenax-TA-containing and Tenax-TA-free treatments (32.4 ± 7.2 and 38.3 ± 0.6 μmoles respectively) and had the slowest rate of toluene degradation (0.1 μmoles/day). This corresponded to 77% stoichiometric yield in these microcosms (Table 3). No methane was produced in any of anoxic microcosms, even in no electron acceptor-added treatments which could have facilitated methanogenic activity.

### 3.3. Microbial Community Analysis

DNA recoveries from sessile (Tenax-TA pouch-associated) samples were greater on average than those recovered from planktonic samples (Table S3 and Figure S4). Sessile fractions on average yielded 1.40 ± 1.00 ng/μL (*n* = 30) with a maximum of 12.3 ng/μL. However, several sessile samples yielded DNA concentrations too low to quantify. All planktonic samples had quantifiable DNA recoveries (maximum 0.48 ng/μL, on average 0.15 ± 0.02 ng/µL; *n* = 63); this represented a statistically significant difference from sessile DNA recoveries (*p*-value ≤ 0.05). Trends in DNA recoveries followed that of the energetics of the electron-accepting processes, where aerobic treatments yielded the most DNA and sulfate-reducing/no electron acceptor-added treatments yielded the least amount of DNA. Concentrations were normalized to the amount of material extracted (liquid or solid; see Table S3).

**Figure 4:**
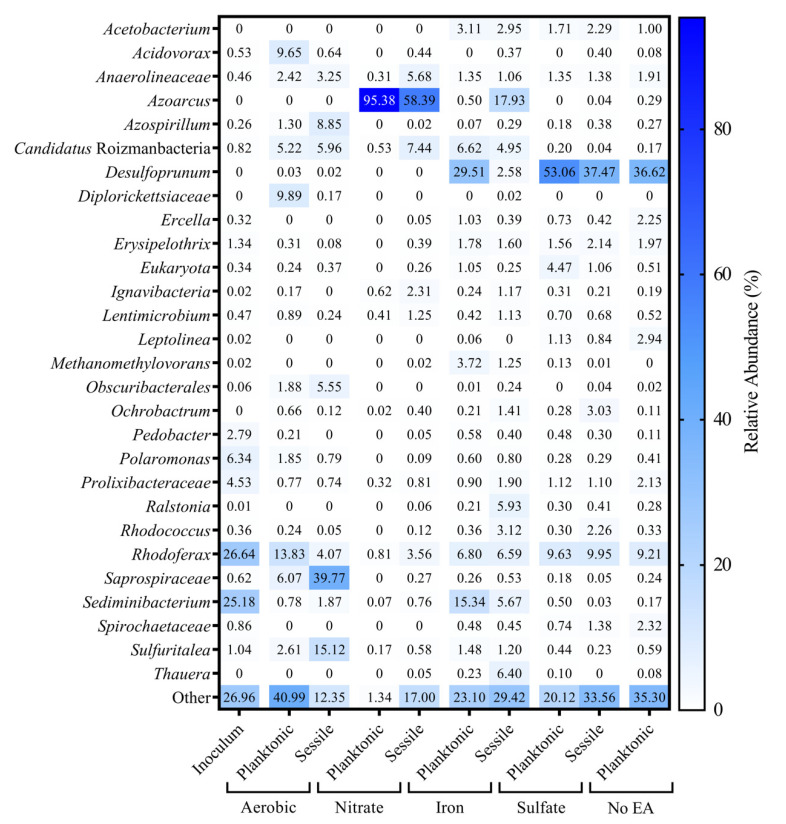
The most abundant microorganisms from each hydrocarbon-amended, Tenax-TA pouch-containing microcosms by treatment as determined using 16S rRNA gene amplicon sequencing. Total reads of three replicates were averaged and are displayed as percent relative abundance. Taxa that did not make comprise the most abundant (generally top five to ten most relative abundant) from each treatment are grouped as ‘Other’. Sessile samples from the no electron acceptor-added treatment could not be amplified through PCR and thus are not included.

The composition of microbial communities from Tenax-TA containing, hydrocarbon-amended treatments diverged from the initial inoculum over the course of the experiment (Figure 4). The groundwater inoculum was primarily composed of *Sediminibacterium* and *Rhodoferax.* The aerobic microcosms became enriched in taxa such as *Rhodoferax, Saprospiraceae,* and *Sulfuritalea.* Nitrate-reducing microcosms were dominated to a large extent by *Azoarcus* with a smaller proportion of *Candidatus* Roizmanbacteria. Communities from iron(III)-amended incubations varied considerably, consisting of taxa identified as *Desulfoprunum, Sedminibacterium, Azoarcus, Rhodoferax,* and *Candidatus* Roizmanbacteria. Sulfate-reducing and the no electron acceptor-added communities were similar to each other, with *Desulfoprunum* in highest relative abundance (36–53%) and *Rhodoferax* in second highest relative abundance (9–10%; Figure 4).

**Figure 5:**
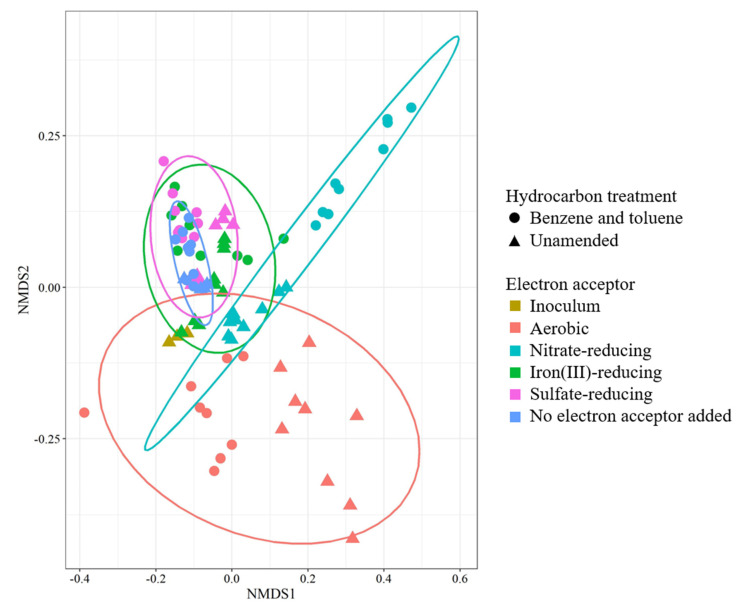
NMDS analysis of 88 microbial communities (microcosms) as analyzed by 16S rRNA gene sequencing. Hydrocarbon-amended treatments are denoted by closed circles (●), while unamended treatments are indicated by closed triangles (▲). Analyses were completed in R.

The primary selective pressure driving community divergence in this study appeared to be the electron-accepting condition, as different redox potentials resulted in distinctive clustering compared to hydrocarbon treatment for most conditions (Figure 5). There was slight grouping within aerobic and nitrate-reducing treatments based on the presence or absence of hydrocarbons, while iron(III)-reducing, sulfate-reducing, and no electron acceptor-added treatments all clustered similarly regardless of the presence of hydrocarbons.

### 3.4. Visualization of Tenax-TA

**Figure 6:**
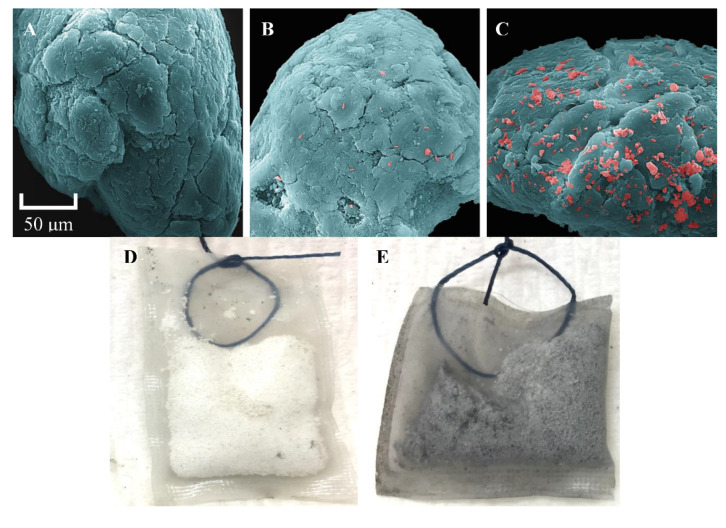
Microbially-colonized Tenax-TA. Panels A–C: false coloured scanning electron micrographs of microbe-colonized Tenax-TA beads retrieved from aerobic incubations. (**A**) The sterile control, (**B**) the live, hydrocarbon-free control, and (**C**) the live, hydrocarbon-amended control. The Tenax-TA bead in panel C has visible microorganisms adhering to its surface while beads in panels A and B show little to no microbial colonization. Panels D and E: Tenax-TA pouches recovered from aerobic microcosms after 80 days of incubation. (**D**) A Tenax-TA pouch from a live, hydrocarbon-free microcosm and (**E**) a Tenax-TA pouch from a live, hydrocarbon-amended treatment. The **Table 4.** Discussion.

Scanning electron microscopy (SEM) was used to visualize microorganisms colonized on the Tenax-TA beads following 80 days of incubation. A single Tenax-TA bead was magnified to see the overall arrangement of microorganisms. In the sterilized control (Figure 6A), no microorganisms are visible on the surface of the beads. In the no-hydrocarbon, live control (Figure 6B), few microorganisms are observed on the surface of the bead. On the live, hydrocarbon-amended treatment (Figure 6C), numerous flaky structures that are believed to be biofilms are visible (structures larger than single bacterial cells), which were not observed on the sterilized or no-hydrocarbon treatments. The presence of hydrocarbons appeared to be necessary for microbial colonization (Figure 6D,E); this is supported by the hydrocarbon degradation observed (Figure S3), notable differences in the composition of microbial communities between sessile and planktonic fractions (Figure 4), and distinct grouping of samples from hydrocarbon-amended and unamended treatments (Figure 5).

### 4.1. Matrix Effects

Tenax-TA is an inert resinous polymer that forms porous beads. It is used in many industrial and analytical applications to purge and trap sorbent chemicals, particularly those with four or more carbons [31]. Its low affinity for water makes it an ideal matrix to reversibly trap airborne volatile organic compounds (VOCs) and aqueous environmental pollutants [50,51]. To the authors’ knowledge, Tenax-TA has not previously been tested or used as a growth surface for microorganisms.

Biochars such as T-carbon and diatomaceous earth (DE) have similar hydrocarbon adsorption properties to Tenax-TA. Highly pyrolyzed biochars (like graphene) and those that were acid treated demonstrated strong but reversible gas adsorption kinetics for both benzene (75–91%, [52]) and toluene (93–98%, [53]). Sheshdeh et al. found that nickel oxide-modified diatomaceous earth adsorbed up to 98% of benzene and 97% of toluene (compared to 74% and 71% from unmodified diatomaceous earth, respectively [54,55]). Raw DE is capable of adsorbing all BTEX hydrocarbons; studies have found in a BTEX mixture, xylenes were more readily sorbed to DE while benzene sorbed the least [56]. Previous findings have shown that Tenax-TA is capable of adsorbing approximately 78% of benzene and 99% of toluene [46], which is comparable albeit slightly higher than our abiotic results (53–77% adsorption; Figure S1).

Biochars generally have high surface areas (up to 340 m^2^/g compared to Tenax-TA’s 35–40 m^2^/g) but are noted to have basic pH in water (9–12), which may have discouraged acidophilic or neutrophilic microorganisms from associating with this material in our field test [57,58]. DE varies widely in its particle size due to its irregular shape and biotic origins, and ranges in surface area from 4 m^2^/g (untreated) up to 50 m^2^/g when decarbonated through acid treatment [59,60]. In a study of DE as a bacterial delivery vehicle, the authors found hollow inner structures within DE that sheltered bacteria, as well as some that sorbed to the surface of the material [61]. This may be one reason why DE materials yielded high DNA recoveries in our field experiment (Figure 1). We found that diversity capture was inversely correlated with DNA recovery for all materials tested, but that high surface area positively correlated with DNA recovery. Overall, Tenax-TA captured the highest diversity of microbial taxa, indicating its usefulness to trap microorganisms in field environments. Sorption of aromatic hydrocarbons was a secondary benefit that was shared between the three materials tested and helped to facilitate capture of putative hydrocarbon degraders.

### 4.2. Biodegradation of Hydrocarbons

Microorganisms within aerobic treatments were able to degrade benzene, and they did so within 14 days of incubation (Figure 3). In contrast, microbial communities incubated anoxically failed to oxidize any benzene over 80 days of monitoring (Figure S3). Given the levels of benzene detected at the Stony Plain site in 2016 (8.39 mg/L [33]), we hypothesized that anaerobic benzene degradation would occur due to its historical exposure to the indigenous microbial population. However, this was not realized within the 80 day incubation period across the anaerobic conditions tested. It has been observed in the few studies that successfully demonstrated anaerobic benzene degradation that long lag times (upwards of 300 days) can be necessary, while many other microcosm studies failed to achieve benzene degradation even after long incubation times [62,63,64]. In the present work, additional incubation time may have been required to observe benzene biodegradation under anoxic conditions.

In contrast to benzene, toluene biodegradation occurred under all experimental conditions (Table 3), with initial lag times generally corresponding to the strength of the associated redox potential (Figure S3). It has been suggested that toluene can act as an inhibitor of benzene biodegradation in anaerobic co-culture studies, which may be another reason that benzene persisted in the microcosms [64]. Across all the conditions tested, the presence of Tenax-TA did not appear to stimulate or inhibit toluene degradation. Microorganisms within live microcosms with and without Tenax-TA consumed toluene at approximately the same rate (Table 3). We hypothesize that hydrocarbon-degrading microorganisms were recruited to the Tenax-TA pouches (Figure 6). However, they were also present planktonically in the liquid phase and were able to readily degrade solubilized aromatic substrates under both conditions.

### 4.3. Mineralization Analysis

In the aerobic incubations, we observed a significant increase in CO_2_ produced from hydrocarbon-degrading microorganisms associated with Tenax-TA compared to incubations without Tenax-TA (Table 3). This amounted to 1.1 μmoles/day of CO_2_ produced from hydrocarbon-amended Tenax-TA microcosms compared to 0.6 μmoles/day in those without Tenax-TA. Differentiated sessile microorganisms living in a biofilm may be a reason for such a marked difference in substrate mineralization, or residual small hydrocarbons that were soluble in the inoculum groundwater may have adsorbed to the Tenax-TA material, in turn increasing their bioavailability. It may also be possible that VOCs present in the air adsorbed to the Tenax-TA material before the pouches were placed in the microcosms, resulting in increased carbon compounds available to the microorganisms [65]. As this trend in higher CO_2_ production was not observed in the sterilized controls, we assume that CO_2_ production was the result of biotic processes. No aromatic hydrocarbons or small organic acids (acetate, butyrate, or propionate) were detected during the initial water chemistry screening, so the exact reason for the varied CO_2_ production remains uncertain.

Nitrate and sulfate reduction did not differ in their extent between microcosms with or without Tenax-TA (Table 3). Iron(III)-reducing microcosms, however, demonstrated unusual patterns of iron(II) production. Iron(II) production was substantial in Tenax-TA-free treatments, but nearly negligible in those containing Tenax-TA (Table 3). Tenax-TA seemed to inhibit iron reduction in this experiment, despite equivalent rates of toluene degradation regardless of Tenax-TA presence. This may be due to the low solubility of Fe(OH)_3_—compared to electron acceptors like O_2_, nitrate, and sulfate—which encouraged microorganisms to associate with the settled iron(III) at the bottom of the microcosms rather than with the hydrocarbon-associated Tenax-TA. A more soluble form of iron(III) could have resulted in better growth and adhesion of microorganisms to Tenax-TA. It is also worth noting that apparent iron sulfide was formed in the iron(III)-reducing, sulfate-reducing, and no electron acceptor-added hydrocarbon-amended microcosms, as the sediment eventually turned black in the live treatments under all of these conditions. This likely resulted from a combination of a reduction in natively present (or exogenously added) iron and sulfate (measured at 1.8 and 0.7 mM in the inoculum, respectively) and hydrogen sulfide production from sulfate reduction, which together precipitated black iron sulfide (visibly observed, not measured).

### 4.4. Microbial Community Analysis

Using Tenax-TA provided a unique opportunity to characterize the microorganisms growing in sessile, aromatic hydrocarbon-associated environments and compare them with associated planktonic communities. The chemical and physical properties of Tenax-TA meant that the hydrocarbons added were largely adsorbed and concentrated on the surface of the material, creating a micro-environment of high local concentration within the microcosm (Figure 6). Microorganisms detected were presumed to not only colonize the Tenax-TA material but also be involved in the metabolism of aromatic hydrocarbons. This speaks to the importance of sessile versus planktonic metabolism in hydrocarbon biodegradation. We observed distinct differences in the microbial communities associated with Tenax-TA in the presence of hydrocarbons compared to the unamended treatments. As such we can infer that there is a large range in metabolic capacity within the indigenous subsurface groundwater microorganisms at the field site examined.

Sequencing analysis of the groundwater prior to its use as the inoculum revealed a native microbial community with many taxa known to be associated with hydrocarbon environments or hydrocarbon-degrading cultures (Figure 4). *Sediminibacterium* (some species of which are facultatively aerobic and associated with degradation of complex carbon compounds [66,67]) and *Polaromonas* (an aerobe believed to be involved in in situ hydrocarbon degradation [68,69]) have previously been detected in cold-climate, hydrocarbon-contaminated environments. *Rhodoferax* is a facultative aerobe with some species capable of iron reduction [70] and is also commonly found in many contaminated sites [71,72]. After 80 days of incubation under aerobic conditions, the sessile communities within hydrocarbon-amended microcosms became enriched in *Saprospiraceae* (39.8% relative abundance) and *Sulfuritalea* (15.1% relative abundance). Planktonic fractions from the same incubations were not similarly enriched in these two taxa. The absence of these taxa from hydrocarbon-free treatments (Figure S5) supports the hypothesis that they are involved in hydrocarbon degradation rather than colonization or adhesion. While *Saprospiraceae* has been implicated in the biodegradation of complex organic compounds [73], it has not previously been associated with hydrocarbon degradation. *Sulfuritalea* on the other hand is commonly found in association with freshwater hydrocarbon contamination. Members of this genus are known to degrade aromatic compounds like benzoate, and have diverse metabolic abilities to use oxygen, arsenate, or sulfur as a terminal electron acceptor [74].

Nitrate-reducing, hydrocarbon-amended microbial communities became enriched in *Azoarcus* (Figure 4) in both the sessile and planktonic phases. Several members of *Azoarcus* are known hydrocarbon degraders [75,76]. The relative abundance of *Azoarcus* in the planktonic fraction (95.4%) outweighed its presence in the sessile fraction (58.4%). Hydrocarbon-free nitrate-reducing communities were very diverse, with 66.3% of taxa representing 0.2% or less of the total community composition (Supplementary Figure S6). Iron-reducing, hydrocarbon-amended planktonic communities were mostly dominated by *Desulfoprunum* (29.5%; Figure 4). As iron(II) production was largely limited in Tenax-TA containing microcosms (Table 3), it is likely the Tenax-TA-associated microenvironment was dominated by sulfate reduction rather than iron(III) reduction. In fact, the iron(III)-treated microbial communities shared many taxa in common with the sulfate-reducing and no electron acceptor-added treatments, and typical iron(III)-reducing hydrocarbon-degraders like *Geobacter* were only found in small abundances (less than 3%, and therefore are part of Other in Figure 4). In sulfate-reducing treatments, *Desulfoprunum* was also present but at higher relative abundances (53.1% planktonic and 37.5% sessile). *Desulfoprunum benzoelyticum* (a benzoate oxidizer) cannot use Fe(OH)_3_ as an electron acceptor but instead uses sulfate [77]. As indicated above, sulfate from the native groundwater was likely used by this taxon in the iron(III)-amended treatments. *Desulfoprunum* reduces sulfate to sulfide, which at high concentrations can be inhibitory to acetate metabolism [77]. In the no electron acceptor-added microcosms, we were unable to obtain PCR amplifiable DNA from the Tenax-TA traps and therefore could not obtain information regarding the composition of the sessile communities. As shown in Figure 4, the microbial communities from the planktonic, no electron acceptor-added and the sulfate-reducing microcosms were very similar in taxonomic distribution with *Desulfoprunum* the most abundant (36.6%) followed by *Rhodoferax* (9.2%), both of which fall within the relative taxonomic abundance observed in sulfate-reducing microcosms. The lack of methane production in these microcosms was supported by the absence of methanogenic taxa such as *Methanosaeta* and *Methanoculleus* commonly found in toluene-degrading, methanogenic communities [10].

Aerobic, nitrate-reducing, and iron(III)-reducing treatments were all moderately enriched (5–7%) in the candidate phylum Roizmanbacterium. This is a relatively recently discovered taxon believed to be facultatively anaerobic, involved in interspecies carbon transfer of small fatty acids, and important in overall carbon flow in groundwater systems [78]. It also possesses genes for oxidation of various carbon sources including necromass and cellulose. As a member of the Candidate Phylum Radiation (CPR) superphylum, many of which are symbionts or exist in a sessile form, *Candidatus* Roizmanbacterium possibly played a role in attachment or adherence within our microcosm system [78].

Aerobic communities diverged in composition considerably from the initial inoculum, as shown in the NMDS analysis (Figure 5). There was distinct clustering of hydrocarbon-amended communities compared to those that were hydrocarbon-unamended. This indicates selection for different populations of microorganisms (i.e., putative hydrocarbon degraders) as a result of the hydrocarbon treatment. This trend was also readily apparent in the nitrate-reducing communities, where hydrocarbon-amended communities diverged greatly from the hydrocarbon-free communities. This was likely due to the substantial increase in the relative abundance of *Azoarcus,* which dominated all nitrate-reducing hydrocarbon-containing treatments. Communities from iron(III)-reducing, sulfate-reducing, and no electron acceptor-added microcosms grouped closely with each other and possessed more similar microbial taxa (Figure 4). Assuming an anoxic state within the subsurface and with minimal input of new nutrients, the microbial communities of the no electron acceptor-added control microcosms would most closely match that which we would expect to see in the field.

## 5. Conclusions

Tenax-TA was an effective matrix to trap microorganisms in groundwater systems for analysis when exposed to aerobic, nitrate-reducing, or sulfate-reducing conditions. Iron-reducing treatments resulted in poor DNA recoveries, but this could be due to the insoluble form of iron that was used and the predominance of sulfate reduction occurring in these microcosms instead. This study revealed that biomass and DNA recoveries overall correlated with the strength of the associated electron acceptor, the presence of carbon substrates, and the length of experimental incubation. Ecological analysis of community data indicated that while the exact taxonomic composition could change depending on hydrocarbon amendment, the overall diversity of the communities was not impacted by the presence of Tenax-TA. For use in our prototype water quality monitoring device, Tenax-TA is an efficient, passive sampling matrix for trapping putative hydrocarbon-degrading microorganisms with the added benefits of its hydrocarbon adsorption properties.

## Figures and Tables

**Table 1:** Conditions and various controls of experimental microcosms. Each combination was conducted under either aerobic, nitrate-reducing, iron(III)-reducing, or sulfate-reducing conditions. A fifth condition received no additional electron acceptor to examine the effect of endogenous electron-accepting processes.

Electron Acceptor	Matrix	Amendment	Treatment
One of: 313 mM O_2_, 10 mM NO_3_^−^, 30 mM Fe(OH)_3_, 10 mM SO_4_^2−^, or none added	Tenax-TA	5.6 μmoles benzene + 4.7 μmoles toluene	Live
			Heat killed
		Unamended	Live
			Heat killed
	No Tenax-TA	5.6 μmoles benzene + 4.7 μmoles toluene	Live
			Heat killed
		Unamended	Live
			Heat killed

**Table 2:** Stoichiometric equations for the complete metabolism of benzene and toluene under the experimental electron-accepting conditions surveyed. Depletion of NO_3_^−^ and SO_4_^2−^ (from nitrate and sulfate-reducing treatments, respectively) and accumulation of CO_2_ (from aerobic treatments), Fe^2+^ (from iron-reducing treatments), and CH_4_ (a possible product from the no EA-added treatments) were monitored in this microcosm study and results are reported in Table 3.

Electron Acceptor (Oxidized/Reduced)	Stoichiometric Equation	Source
O_2_/CO_2_	C_6_H_6_ + 7.5O_2_ → 6CO_2_ + 3H_2_O	[47]
	C_7_H_8_ + 9O_2_ → 7CO_2_ + 4H_2_O	[48]
NO_3_^−^/N_2_	C_6_H_6_ + 6NO_3_^−^ → 6HCO_3_^−^ + 3N_2_	[13]
	C_7_H_8_ + 7.2NO_3_^−^ + 7.2H^+^ → 7CO_2_ + 7.6H_2_O + 3.6N_2_	[48]
Fe^3+^/Fe^2+^	C_6_H_6_ + 18H_2_O + 30Fe^3+^ → 6HCO_3_^−^ + 30Fe^2+^ + 36H^+^	[13]
	C_6_H_6_ + 21H_2_O + 36Fe^3+^ → 7HCO_3_^−^ + 36Fe^2+^ + 43H^+^	[7]
SO_4_^2−^/H_2_S	C_6_H_6_ + 3.75SO_4_^2−^ + 3H_2_O → 3.75HS^−^ + 6HCO_3_^−^ + 2.25H^+^	[13]
	C_7_H_8_ + 4.5SO_4_^2−^ + 3H_2_O → 2.25H_2_S + 2.25HS^−^ + 7HCO_3_^−^ + 0.25H^+^	[8]
CO_2_/CH_4_	C_6_H_6_ + 4.5H_2_O → 3.75CH_4_ + 2.25CO_2_	[49]
	C_7_H_8_ + 5H_2_O → 4.5CH_4_ + 2.5CO_2_	[49]

**Table 3:** Predicted and actual hydrocarbon transformation yields in microcosms with (+ Tenax-TA) and without (- Tenax-TA) Tenax-TA under various electron-accepting conditions. Predicted product yields or electron acceptor consumption were calculated using the stoichiometric equations shown in Table 2. Actual yields/consumptions were measured as described in Materials and Methods and averaged from triplicate microcosms; error bars indicate standard error of the mean. An unpaired, two-tailed *t*-test was calculated to determine statistically significant differences between the product yields from Tenax-TA and no Tenax-TA-treated microcosms.

Redox Condition	Chemical Monitored	Total Hydrocarbons Consumed (μmoles)	Degradation Rate (μmoles/Day)	Predicted Yield or Consumption (μmoles)	Actual Yield or Consumption (μmoles)		*p*-Value
					+ Tenax-TA	− Tenax-TA	
Aerobic	CO_2_	31.0	0.4	199.9	97.9 ± 0.7	55.0 ± 2.0	**
Nitrate reducing	NO_3_^−^	18.8	0.2	135.4	91.8 ± 5.5	113.2 ± 4.6	*
Iron(III) reducing	Fe^2+^	14.1	0.2	507.6	35.9 ± 1.7	267.0 ± 68.4	ns
Sulfate reducing	SO_4_^2−^	9.4	0.1	42.3	32.4 ± 7.2	38.3 ± 0.6	ns
No EA added	CH_4_	14.1	0.2	63.5	0.0	0.0	ns

ns = not significant, * *p*-value ≤ 0.05, and ** *p*-value ≤ 0.01.

## Data Availability

The data presented in this study are available within the article and the associated supplementary materials.

## Supplementary Materials

The following are available online at https://www.mdpi.com/2076-2607/9/1/90/s1, Table S1. DNA recoveries from the initial survey of trapping materials efficacy in water and soil to loosely approximate a sampling well with exposed soil (5 g soil in 30 mL sterile DI water). Fertilizer (Miracle-Gro Garden Feeder, 28-8-16) was added at 0.33 g/L to one treatment to enhance growth. Matrix materials included zeolite (molecular sieve, 8–12 mesh, 3Å, 208582; Sigma Aldrich, Oakville, Canada), activated carbon (CAS 7440-44-0, L16334; Alfa Aesar, Haverhill, MA, USA), Mat540 (porous 30 μm silica microspheres; Materium Innovations, Ithaca, NY, USA), diatomaceous earth (Red Lake Earth, Kamloops, Canada), and ZMM^®^ T-carbon (biochar from 2 mm woody feedstock; Canada Minerals Corp., Peachland, BC, Canada). DNA was extracted from two replicates of each material following 6 days of incubation at room temperature in the dark and removal of excess soil by gentle rinsing with sterile DI water. No DNA was recovered from activated carbon or Mat540, while only 0.3–0.6 ng/µL was recovered from zeolite. Average DNA recoveries from duplicate extractions are shown. Table S2. DNA extraction concentrations from field trials with diatomaceous earth (DE), T-carbon (TC), and Tenax-TA (TA). Trap samplers were deployed into a hydrocarbon-contaminated aquifer and recovered in one-month intervals for a total of three months. Recovered and extracted DNA concentrations are provided for each replicate. Some samples had DNA concentrations higher than the detection limit (>60 ng/μL) of the instrument and are reported as “too high” or TH. Values for Shannon and Simpson diversity indices were computed in R using vegan. Table S3. Normalized DNA recoveries from experimental Tenax-TA incubations, comparing incubations with or without hydrocarbons (HCs), with or without Tenax-TA, and the fraction the sample was collected from (planktonic or sessile). Extracted DNA concentrations were normalized based on the amount of starting material (0.13 g for sessile samples, 5 mL for planktonic samples). DNA recoveries that were too low to quantify (<0.05 ng/μL) are denoted as NA. Figure S1. Hydrocarbons measured in abiotic sorption tests one day after addition. Toluene sorption to Tenax-TA represented 77% of available hydrocarbons (without Tenax-TA), while benzene sorption represented 53%. Asterisks represent statistically significant differences as calculated by an unpaired two-tailed *t*-test (** *p*-value ≤ 0.01, and *** *p*-value ≤ 0.001). Figure S2. Design of experimental microcosms. Glass serum bottles were sealed with the Tenax-TA filled pouch suspended into the aqueous phase. Groundwater was added; associated sand settled to the bottom over time. Aerobic treatments received air as the headspace while anoxic treatments were flushed with a headspace of N_2_ gas. Figure S3. Toluene and benzene degradation profiles in aerobic (panels A and B), nitrate-reducing (C and D), iron(III)-reducing (E and F), sulfate-reducing (G and H), and no electron acceptor-added (I and J) microcosms, respectively, over 80 days of incubation in the presence and absence of Tenax-TA. Both live incubations and heat-killed controls (HK) were established. Error bars represent the standard error of the mean of three replicates. Breaks in plot lines represent re-amendment of hydrocarbons after a time point where all previously added hydrocarbons had been consumed. Figure S4. DNA recovered from hydrocarbon-amended (+HCs) and -unamended (-HCs) Tenax-TA pouches across different treatments. Tenax-TA samplers from aerobic microcosms with hydrocarbons yielded the most DNA (10.15 ± 1.22 ng/µL) while DNA was below detection limits in iron(III)-reducing microcosms regardless of the presence of hydrocarbons. Error bars represent the standard error of the mean of three replicates. Asterisks represent statistically significant differences as calculated by an unpaired two-tailed *t*-test (** *p*-value ≤ 0.01). Figure S5. Microbial community composition of aerobic microcosms including the groundwater used as the inoculum as well as all treatments and controls. Samples were analyzed through 16S rRNA gene sequencing in triplicate and the averages of that analysis are shown here. Hydrocarbon-amended samples with Tenax-TA (TA) have already been discussed in detail in Figure 4. In the planktonic hydrocarbon-unamended treatments (no HCs), *Candidatus* Nitrocosmicus was found to be the most abundant. Figure S6. Microbial community composition of nitrate-reducing microcosms including the groundwater used as the inoculum as well as all treatments and controls. Samples were analyzed through 16S rRNA gene sequencing in triplicate and the averages of that analysis are shown here. Hydrocarbon-amended samples with Tenax-TA were dominated by *Azoarcus* and have already been discussed in detail in Figure 4. Hydrocarbon-unamended planktonic communities were dominated by *Rhodoferax* and *Candidatus* Roizmanbacteria, while sessile communities were incredibly diverse, with 66% the total relative abundance comprising a variety of taxa each making up less than 0.2% of the overall community.
