# Supplementary material for: The Effect of an Adsorbent Matrix on Recovery of Microorganisms from Hydrocarbon-Contaminated Groundwater
Source: Microorganisms. 2021 Jan 1;9(1):90. doi: 10.3390/microorganisms9010090 (PMC7823327; doi:10.3390/microorganisms9010090)
Supplement: Supplementary file 1 [file microorganisms-09-00090-s001.pdf]

Supplementary Material

The effect of an adsorbent matrix on recovery of microorganisms from hydrocarbon-contaminated groundwater

Nicole M. Taylor [1], Courtney R.A. Toth [2], Victoria Collins [3], Paolo Mussone [3] and Lisa M. Gieg [1,*]

[1] Petroleum Microbiology Research Group, Department of Biological Sciences, University of Calgary, 2500 University Drive NW, Calgary, AB T2N 1N4, Canada; nicole.taylor1@ucalgary.ca (N.M.T.)
[2] Department of Chemical Engineering and Applied Chemistry, University of Toronto, 200 College Street, Toronto, ON, M5S 3E5, Canada; courtney.toth@utoronto.ca (C.R.A.T.)
[3] Applied BioNanotechnology Industrial Research Chair Program, Northern Alberta Institute of Technology, 11762-106 Street, Edmonton, AB T5G 2R1, Canada; ccollins@nait.ca (V.C.); pmussone@nait.ca (P.M.)
* Correspondence: lmgieg@ucalgary.ca (L.M.G.)

3 Supplementary Tables

6 Supplementary Figures

Table S1. DNA recoveries from the initial survey of trapping materials efficacy in water and soil to loosely approximate a sampling well with exposed soil (5 g soil in 30 ml sterile DI water). Fertilizer (Miracle-Gro Garden Feeder, 28-8-16) was added at 0.33 g/L to one treatment to enhance growth. Matrix materials included zeolite (molecular sieve, 8-12 mesh, 3Å, 208582; Sigma Aldrich, Oakville, Canada), activated carbon (CAS 7440-44-0, L16334; Alfa Aesar, Haverhill, USA), Mat540 (porous 30 μm silica microspheres; Materium Innovations, Ithaca, USA), diatomaceous earth (Red Lake Earth, Kamloops, Canada), and ZMM® T-carbon (biochar from 2 mm woody feedstock; Canada Minerals Corp., Peachland, Canada). DNA was extracted from two replicates of each material following 6 days of incubation at room temperature in the dark and removal of excess soil by gentle rinsing with sterile DI water. No DNA was recovered from activated carbon or Mat540, while only 0.3-0.6 ng/μL was recovered from zeolite. Average DNA recoveries from duplicate extractions are shown.

	DNA (ng/μL)				
	Zeolite	Activated carbon	Mat540	Diatomaceous earth	T-carbon
Positive control (soil + fertilizer)	0.45-0.75	0.0	0.0	6.05-6.15	5.5-7.1
Experimental (soil only)	0.3	0.0	0.0	3.6-4.0	2.0-2.2
Negative control (autoclaved soil)	0.0	0.0	0.0	0.85-0.95	0.9-1.1
Blank (sterile, no soil)	0.0	0.0	0.0	0.0	0.0

Table S2. DNA extraction concentrations from field trials with diatomaceous earth (DE), T-carbon (TC), and Tenax-TA (TA). Trap samplers were deployed into a hydrocarbon-contaminated aquifer and recovered in one-month intervals for a total of three months. Recovered and extracted DNA concentrations are provided for each replicate. Some samples had DNA concentrations higher than the detection limit (>60 ng/µL) of the instrument and are reported as "too high" or TH. Values for Shannon and Simpson diversity indices were computed in R using vegan.

Time (months)	Trap material	Sampling depth (m)	DNA concentration (ng/µL)	Average Shannon diversity	Average Simpson diversity
1	DE	3	1.58-2.92	2.88	0.88
		4	3.35-3.72	2.46	0.84
		5	3.48-6.76	1.77	0.68
	TA	3	3.09-4.16	2.76	0.86
		4	1.07-1.66	2.48	0.83
		5	0.76-0.88	2.69	0.80
	TC	3	3.51-4.43	2.37	0.84
		4	3.39-3.79	1.66	0.64
		5	2.68-3.45	2.34	0.79
2	DE	3	2.06-8.77	2.84	0.90
		4	6.70-8.24	2.70	0.87
		5	4.33-6.25	3.25	0.93
	TA	3	0.88-2.77	2.92	0.88
		4	2.07-5.08	2.97	0.92
		5	3.88-3.99	3.34	0.94
	TC	3	0.42-0.93	2.49	0.83
		4	5.87-7.24	2.63	0.87
		5	4.19-6.32	2.90	0.91
3	DE	3	14.0-22.7	2.59	0.81
		4	5.00-5.81	2.82	0.88
		5	9.42-11.0	3.54	0.95
	TA	3	5.50-9.05	3.46	0.93
		4	2.46-3.97	3.17	0.93
		5	9.49-11.5	3.56	0.95
	TC	3	37.2-TH	3.21	0.91
		4	6.84-7.91	2.83	0.90
		5	13.8-19.6	3.05	0.90

Table S3. Normalized DNA recoveries from experimental Tenax-TA incubations, comparing incubations with or without hydrocarbons (HCs), with or without Tenax-TA, and the fraction the sample was collected from (planktonic or sessile). Extracted DNA concentrations were normalized based on the amount of starting material (0.13 g for sessile samples, 5 mL for planktonic samples). DNA recoveries that were too low to quantify (<0.05 ng/μL) are denoted as NA.

Electron acceptor	HCs	Planktonic/ Sessile	Tenax-TA	Normalized DNA per g sample (ng/μL)			Average DNA (ng/μL)	SEM
				R1	R2	R3		
Inoculum	NA	P	NA	0.335	0.317	0.354	0.335	0.011
O_2	+	P	+	0.076	0.177	0.211	0.155	0.041
	+	S	+	12.300	10.100	8.060	10.153	1.224
	-	P	+	0.062	0.162	0.069	0.098	0.032
	-	S	+	10.600	11.700	8.000	10.100	0.542
	+	P	-	0.276	0.304	0.208	0.263	0.029
	-	P	-	0.138	0.132	0.059	0.110	0.025
NO_3^-	+	P	+	0.179	0.268	0.330	0.259	0.044
	+	S	+	1.060	1.860	3.650	2.190	0.766
	-	P	+	0.090	0.088	0.153	0.110	0.021
	-	S	+	NA	0.057	NA	0.057	0.019
	+	P	-	0.458	0.411	0.481	0.450	0.021
	-	P	-	0.167	0.154	0.147	0.156	0.006
Fe^{3+}	+	P	+	0.077	0.118	0.079	0.091	0.013
	+	S	+	NA	NA	NA	NA	NA
	-	P	+	0.079	0.119	0.115	0.104	0.013
	-	S	+	NA	NA	NA	NA	NA
	+	P	-	0.038	0.132	0.097	0.089	0.027
	-	P	-	0.113	0.212	0.122	0.149	0.032
SO_4^{2-}	+	P	+	0.091	0.041	0.041	0.058	0.017
	+	S	+	NA	NA	0.492	0.492	0.164
	-	P	+	0.079	0.068	0.071	0.072	0.003
	-	S	+	NA	NA	NA	NA	NA
	+	P	-	0.102	0.098	0.093	0.098	0.003
	-	P	-	0.101	0.106	0.086	0.098	0.006
No EA added	+	P	+	0.087	0.072	0.066	0.075	0.006
	+	S	+	NA	NA	NA	NA	NA
	-	P	+	0.116	0.091	0.089	0.099	0.009
	-	S	+	0.132	NA	NA	0.132	NA
	+	P	-	NA	0.111	0.126	0.118	0.008
	-	P	-	0.200	0.183	0.184	0.189	0.006

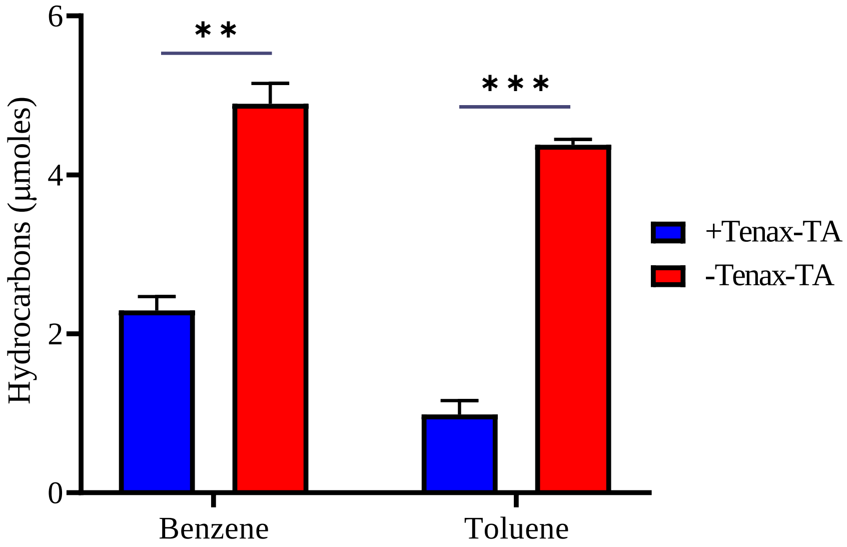

Figure S1. Hydrocarbons measured in abiotic sorption tests one day after addition. Toluene sorption to Tenax-TA represented 77% of available hydrocarbons (without Tenax-TA), while benzene sorption represented 53%. Asterisks represent statistically significant differences as calculated by an unpaired two-tailed *t*-test (** p-value ≤ 0.01, *** p-value ≤ 0.001).

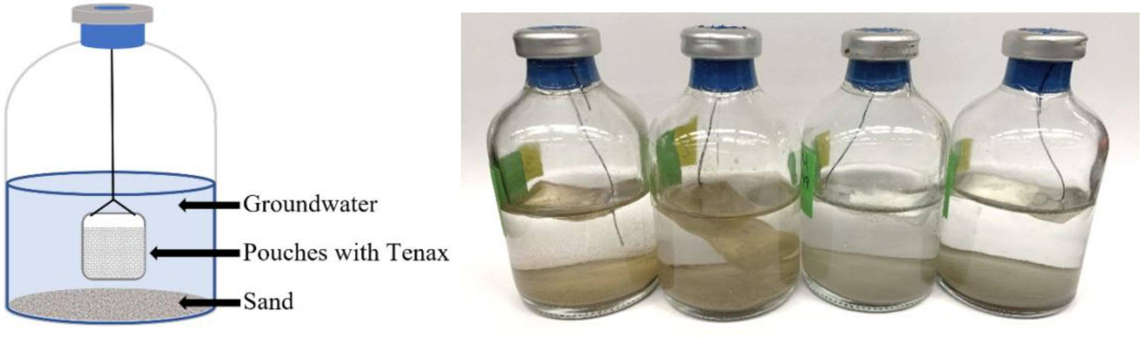

Figure S2. Design of experimental microcosms. Glass serum bottles were sealed with the Tenax-TA filled pouch suspended into the aqueous phase. Groundwater was added; associated sand settled to the bottom over time. Aerobic treatments received air as the headspace while anoxic treatments were flushed with a headspace of N_2 gas.

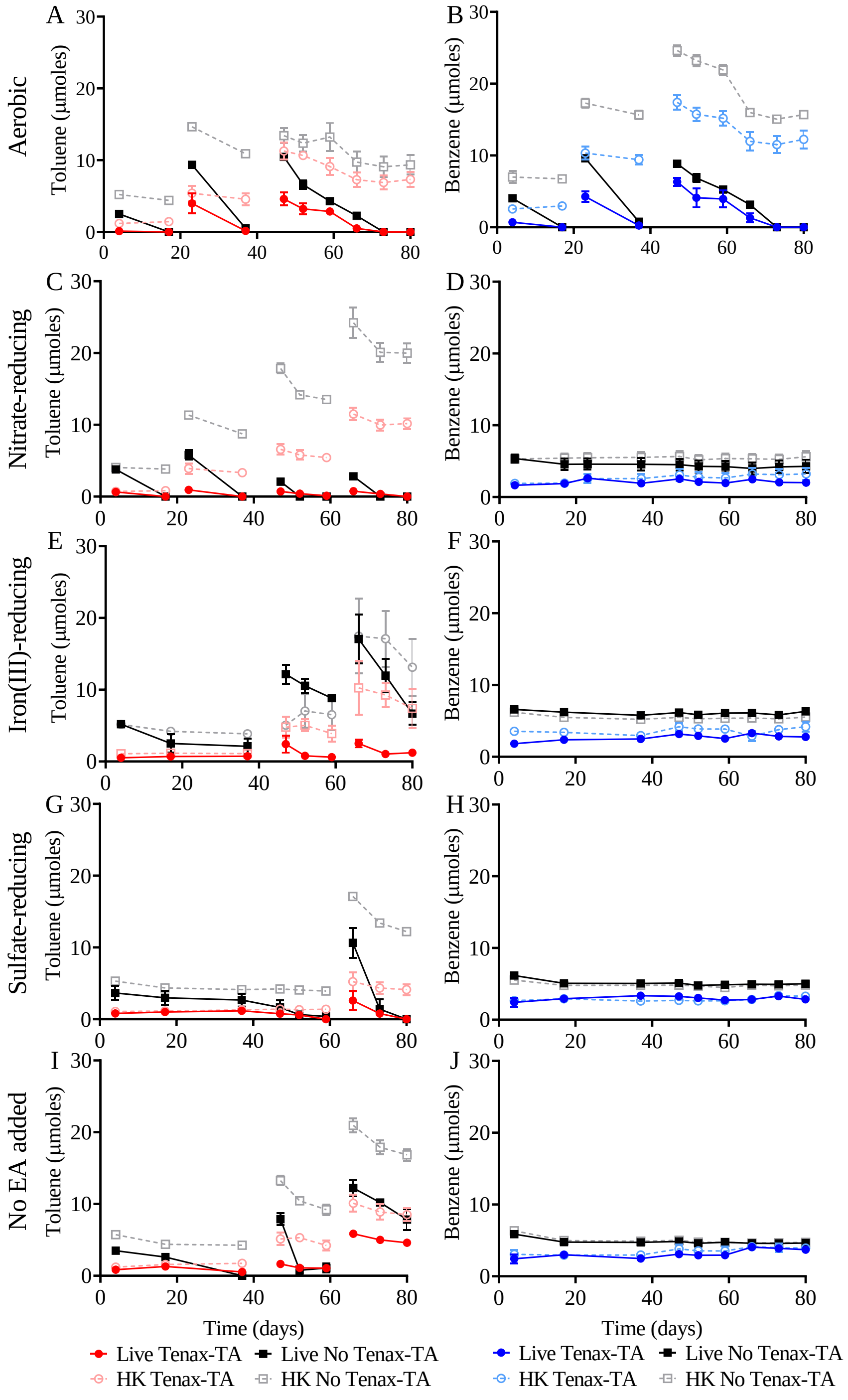

Figure S3. Toluene and benzene degradation profiles in aerobic (panels A and B), nitrate-reducing (C and D), iron(III)-reducing (E and F), sulfate-reducing (G and H), and no electron acceptor-added (I and J) microcosms, respectively, over 80 days of incubation in the presence and absence of Tenax-TA. Both live incubations and heat-killed controls (HK) were established. Error bars represent the standard error of the mean of 3 replicates. Breaks in plot lines represent re-amendment of hydrocarbons after a time point where all previously added hydrocarbons had been consumed.

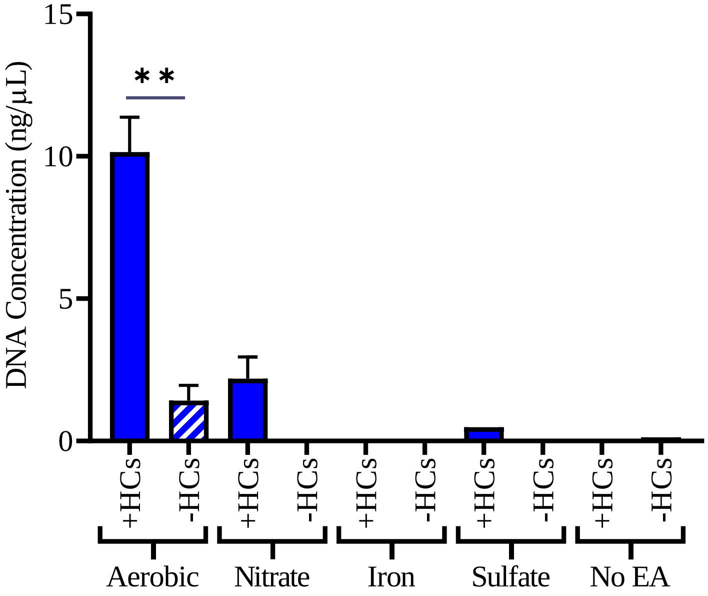

Figure S4. DNA recovered from hydrocarbon-amended (+HCs) and unamended (-HCs) Tenax-TA pouches across different treatments. Tenax-TA samplers from aerobic microcosms with hydrocarbons yielded the most DNA (10.15 ± 1.22 ng/μL) while DNA was below detection limits in iron(III)-reducing microcosms regardless of the presence of hydrocarbons. Error bars represent the standard error of the mean of 3 replicates. Asterisks represent statistically significant differences as calculated by an unpaired two-tailed t-test (** *p*-value ≤ 0.01).

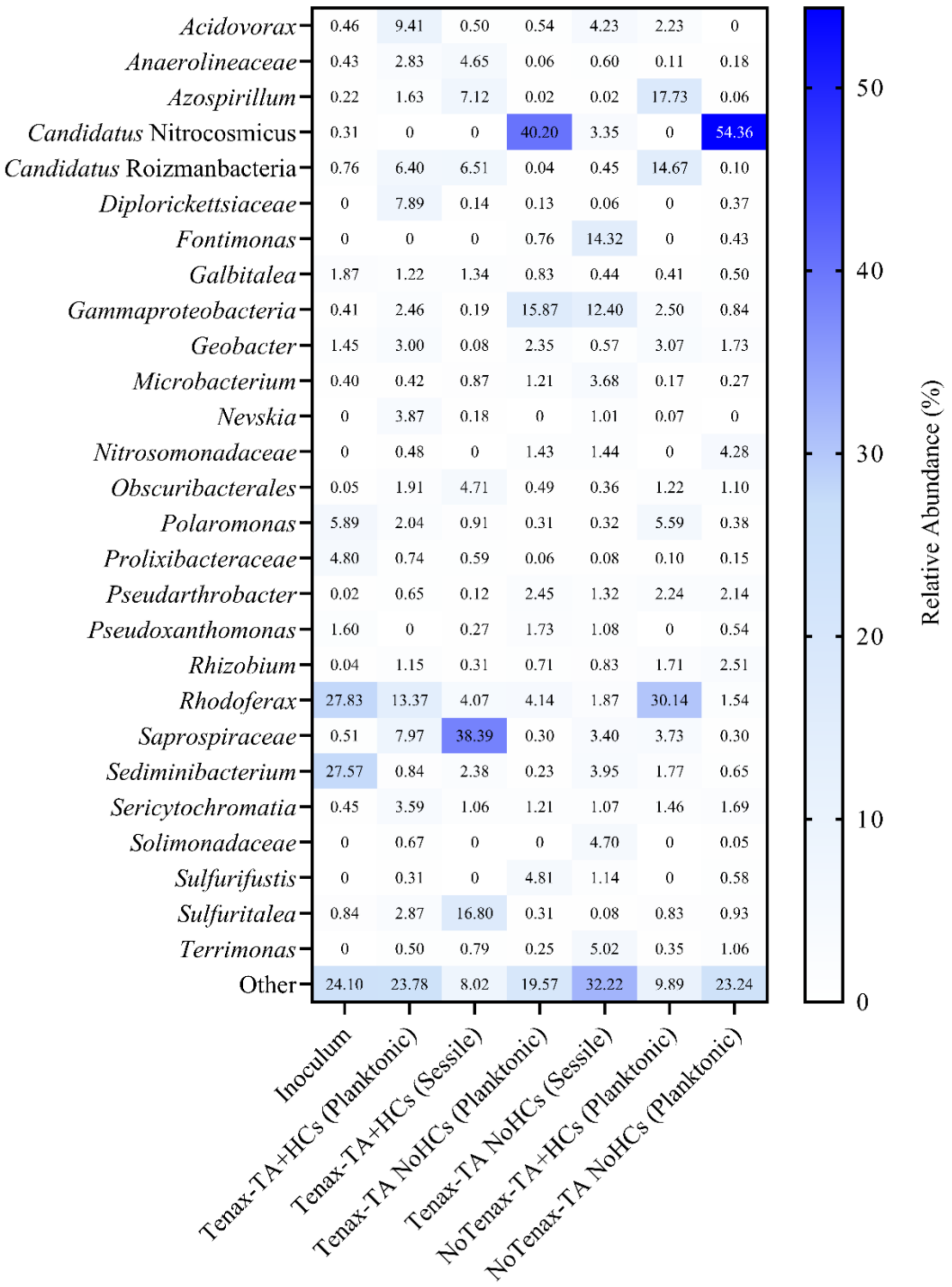

Figure S5. Microbial community composition of aerobic microcosms including the groundwater used as the inoculum as well as all treatments and controls. Samples were analyzed through 16S rRNA gene sequencing in triplicate and the averages of that analysis are shown here. Hydrocarbon-amended samples with Tenax-TA (TA) have already been discussed in detail in Figure 4. In the planktonic hydrocarbon-unamended treatments (no HCs), *Candidatus* Nitrocosmicus was found to be the most abundant.

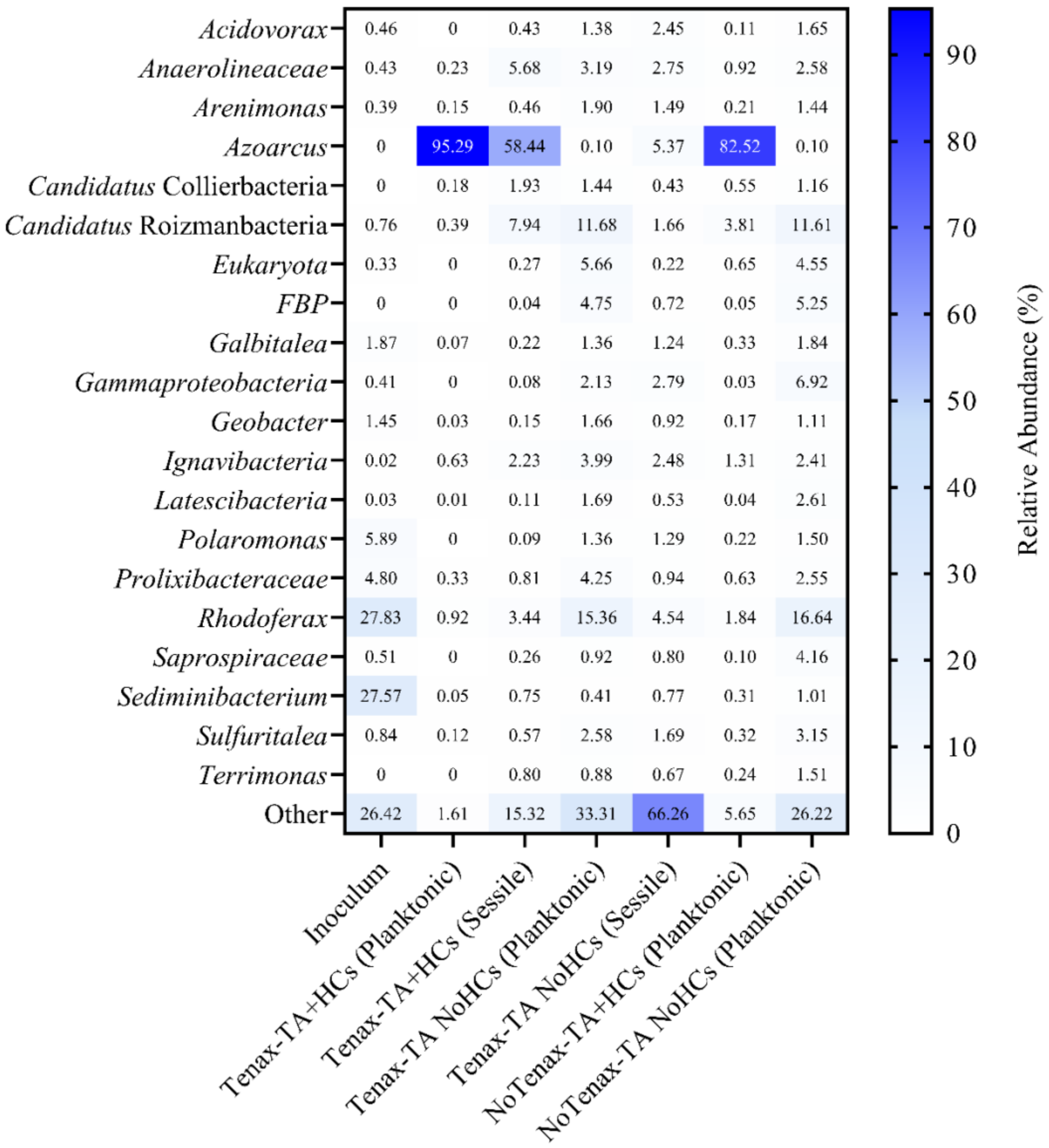

Figure S6. Microbial community composition of nitrate-reducing microcosms including the groundwater used as the inoculum as well as all treatments and controls. Samples were analyzed through 16S rRNA gene sequencing in triplicate and the averages of that analysis are shown here. Hydrocarbon-amended samples with Tenax-TA were dominated by *Azoarcus* and have already been discussed in detail in Figure 4. Hydrocarbon-unamended planktonic communities were dominated by *Rhodoferax* and *Candidatus* Roizmanbacteria, while sessile communities were incredibly diverse, with 66% the total relative abundance comprising a variety of taxa each making up less than 0.2% of the overall community.
